# Omphalomesenteric Ducts and Urachal Remnants: A Retrospective Study and Case Series

**DOI:** 10.7759/cureus.63877

**Published:** 2024-07-05

**Authors:** Aya Tanaka, Takayuki Fujii, Hiroto Katami, Ryou Ishikawa, Reiji Haba, Ryuichi Shimono

**Affiliations:** 1 Pediatric Surgery, Kagawa University, Takamatsu, JPN; 2 Diagnostic Pathology, Kagawa University Hospital, Takamatsu, JPN

**Keywords:** umbilical problems, laparoscopic approach, urachal remnant, surgical management, omphalomesenteric duct remnant

## Abstract

Purpose: The management strategies for umbilical disorders remain undefined. This study aims to review our experience and propose a management algorithm for symptomatic urachal and omphalomesenteric duct anomalies.

Methods: We retrospectively reviewed medical charts between January 2013 and September 2017 of 28 patients with clinical concern for umbilical disorders, out of which 10 were diagnosed with omphalomesenteric duct remnants (OMDR) or urachal remnants (UR). We assessed patients’ sex, age at operation, initial presentation, imaging findings, surgical approach, histopathological findings, and prognostic outcome.

Results: Among 10 patients with OMDR or UR, initial presentations were omphalitis in four, umbilical discharge in three, abdominal pain in two, and umbilical mass in one. Ultrasonography (US), computed tomography (CT), magnetic resonance imaging (MRI), and voiding cystourethrography were performed in 10, seven, three, and four patients, respectively. Transumbilical extraperitoneal excision from a small expanded umbilical incision and laparoscopic approach combined with transumbilical excision was performed in eight and two patients, respectively. Postoperative wound infection occurred in 10% of patients.

Discussion and conclusion: Ultrasonography was mostly used as an initial diagnostic modality, and in cases in which there were signs of infection, they were drained adequately; CT/MRI was chosen for further evaluation of suspicious cases for other complications. Thus, we recommended surgical excision in cases with persistent umbilical disorders. The umbilical approach displays good cosmetic results with easy, complete excision, and the laparoscopic approach could be an excellent diagnostic and therapeutic method for the management of complicated conditions.

## Introduction

Umbilical problems, such as discharge, granulation, and omphalitis, are commonly witnessed in pediatric patients. Thus, an underlying congenital anomaly, the omphalomesenteric duct remnant (OMDR) or urachal remnant (UR), should always be ruled out. The omphalomesenteric duct sometimes has a connection between the yolk sac and primitive midgut, and OMDR occurs if its resorption is incomplete [1]. The urachus is a normal embryonic structure that arises from the allantois, and UR originates from the failure of the obliteration of the allantois. Reportedly, OMDR and UR occur in approximately 2% of the population [2, 3]. Preoperative differentiation between OMDR and UR is difficult, and the diagnosis and management of umbilical problems are sometimes difficult as there is no proper guideline. Thus, this study aims to review our experience and propose a diagnosis and management algorithm for symptomatic urachal and omphalomesenteric duct anomalies.

## Materials and methods

In our study, we retrospectively examined 28 patients who were referred to Kagawa University Hospital, Takamatsu, Japan, due to umbilical disorders between January 2013 and September 2017. Among 18 (64.3%) patients, 10 were clinically diagnosed with umbilical granuloma, three with omphalitis, and five had an unclear diagnosis without surgical intervention. The remaining 10 (35.7%) patients who were diagnosed with OMDR or UR and required surgical intervention were retrospectively examined in this study. Both OMDR and UR were diagnosed by clinical examination and diagnostic imaging. The diagnostic imaging methods used were ultrasonography (US), computed tomography (CT), voiding cystourethrography (VCUG), and magnetic resonance imaging (MRI). All the patients diagnosed with OMDR or UR received surgical excision or correction. We analyzed patients according to sex, age at operation, initial presentation, imaging findings, treatment methods, operative findings, histopathological findings, and prognostic outcome. Histopathology of the resected specimens confirmed the diagnosis of OMDR or UR.

Operative procedure

Eight patients received transumbilical extraperitoneal excision from a small-expanded umbilical incision, which means a reversed omega-shaped infraumbilical incision. Case 4 had laparoscopic exploration due to suspicions of OMRD connected to the intestine and required transumbilical excision. Case 8 had an abscess, and due to the findings of a large lesion on MRI, a laparoscopic approach combined with transumbilical excision was used. A Foley catheter was inserted into the bladder in cases 4 and 8. The first 5 mm of the trocar was placed on the right side of the abdomen by the periumbilical incision as the camera port. The abdominal cavity is inflated with carbon dioxide (CO_2_) gas at 8 mmHg pressure using a 5 mm, 30° rigid camera scope. An additional three ports were inserted into the abdomen under laparoscopic vision in case 8.

This study protocol was approved by the ethics review board of Kagawa University, Takamatsu, Japan (approval no. H29-195).

## Results

In this study, the male-to-female ratio was 5:5, and the age at diagnosis ranged from two days to 22 years (Table 1).

**Table 1:** Patient characteristics F: female; M: male; VCUG: voiding cystourethrography; OMDR: omphalomesenteric duct remnant; UA: umbilical approach; LA: laparoscopic approach

Case	Sex	Age at operation	Initial symptom	Imaging	Final Diagnosis	Operation
1	F	15 days	Umbilical mass	US, CT, VCUG	Urachal cyst	UA
2	M	28 days	Omphalitis	US, CT, VCUG	Urachal sinus	UA
3	M	9 month	Omphalitis	US, CT, VCUG	Urachal cyst	UA
4	M	1 year	Omphalitis	US, CT	Umbilical sinus related to OMDR	UA, LA for exploration
5	M	10 years	Umbilical discharge	US, MRI, VCUG	Urachal sinus	UA
6	F	10 years	Abdominal pain	US, MRI	Urachal sinus	UA
7	F	14 years	Umbilical discharge	US, CT	Urachal sinus	UA
8	F	15 years	Abdominal pain	US, CT, MRI	Urachal cyst	LA
9	F	15 years	Umbilical discharge	US	Urachal sinus	UA
10	M	22 years	Omphalitis	US, CT	Urachal sinus	UA

**Figure 1:**
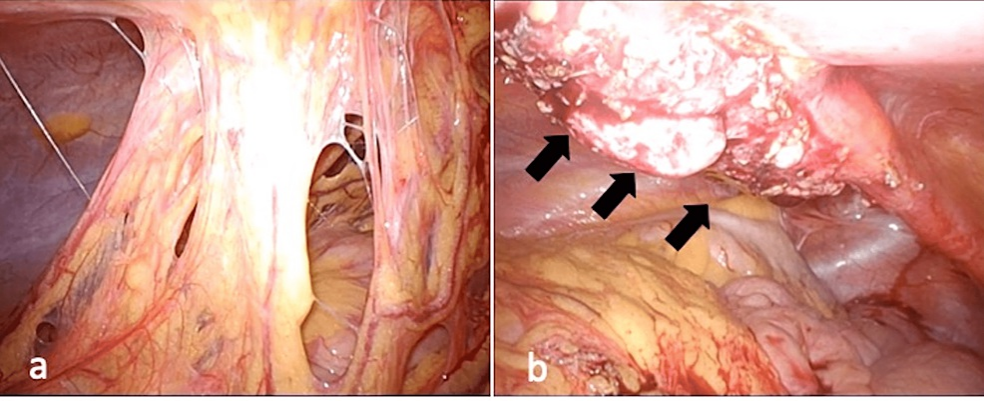
Laparoscopic findings of case 8 a) the omentum was seen adhering to the abdominal wall; b) black arrows showed the cystic urachal remnant.

The initial presentations were as follows: omphalitis in four patients (40%), umbilical discharge in three (30%), and abdominal pain in two (20%). In addition, three patients were treated with drainage and antibiotics for preoperative abscess formation. All 10 patients underwent US; four had VCUG, seven had CT, and three had MRI. Laparoscopic findings showed omentum adhesion and cystic URs in case 8 (Figure 1).

**Figure 2:**
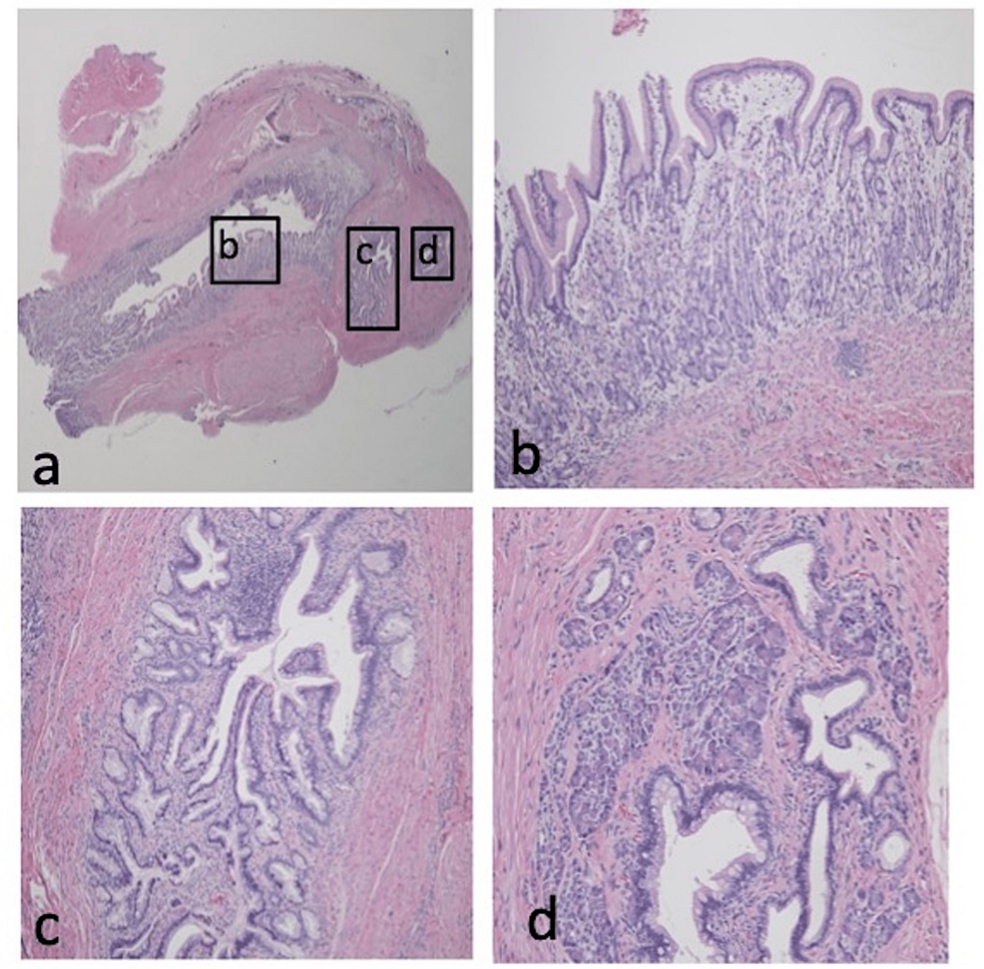
Histopathological findings of case 4 a) hematoxylin and eosin micrographs (×10); b) ectopic gastric mucosa is visible inside the umbilical cyst (×100); c) the small bowel mucosa with villous architecture (×100); d) lobules of pancreatic tissue, comprising acini and islets of Langerhans (×200).

Histopathological examination revealed urothelium in four, scars or granulation in five, and ectopic gastric mucosa with small bowel and pancreatic tissue in one patient (case 4), who exhibited severe and prolonged omphalitis preoperatively (Figure 2).

For the final diagnosis, a urachal cyst was detected in six patients (60%), a urachal sinus in three (30%), and an umbilical sinus occurring as an exceedingly rare variant of OMDR in one (10%). Besides those, we identified no dysplastic changes or malignancies. Furthermore, postoperative wound infection occurred in one (case 6) of 10 patients (10%).

## Discussion

**Figure 3:**
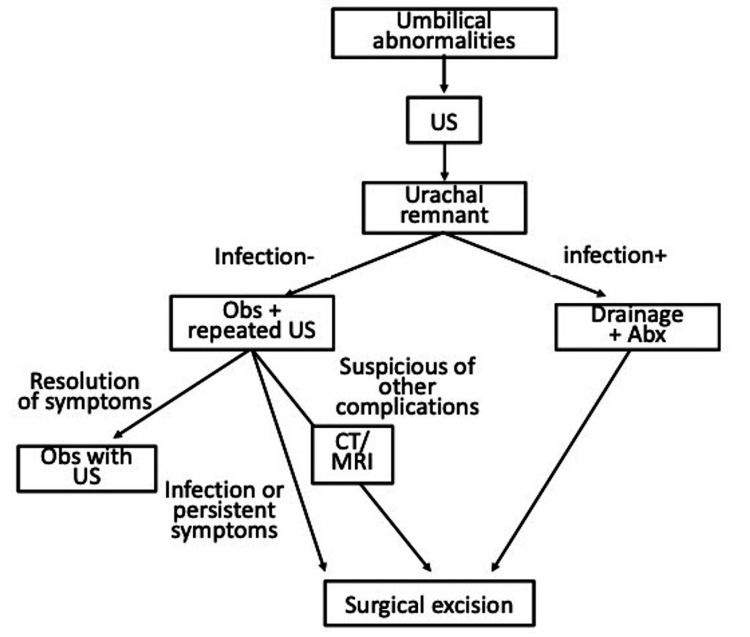
The proposed management algorithm for symptomatic urachal and omphalomesenteric duct anomalies abx: antibiotics; obs: observation

To date, uniform guidelines for the diagnosis and surgical strategy of umbilical problems are lacking. This study proposes a management algorithm for symptomatic umbilical anomalies in pediatric patients (Figure 3).

Despite radiographic assessment, preoperative differentiation of an OMDR from UR might still be difficult. In fact, the final diagnosis might require surgical intervention for the precise determination of the origin [2], and pathological examination is required in resected specimens. In our cohort, US was performed in 100%, CT in 70%, MRI in 30%, and VCUG in 40% of patients. The findings, such as patent urachus, umbilical sinus, and urachal cyst, revealed that US is a useful primary imaging method for the diagnosis of umbilical problems because of its high positive predictive value (83%) and sensitivity (79%) for the diagnosis of OMDR or UR [3]. Although OMDR or UR could be definitively diagnosed by US in most cases, additional radiographic assessments, such as CT, MRI, and/or VCUG because of the precisions, might be necessary for cases with negative or questionable findings by US [4]. In addition, although VCUG could determine the presence of bladder diverticulum, a study established its sensitivity at 5.9% for the diagnosis of UR [5]. Likewise, the utility of VCUG has been questioned previously and is no longer considered one of the standard evaluation methods for urachal anomalies [3, 5]. Besides, CT/MRI could investigate other complicated abnormalities, such as the band connecting to the intestine or other conditions. Given the increased lifetime cancer risk and limited additional information gained, CT should be reserved for ambiguous US results [4, 5]. Based on our experience, CT/MRI successfully detected the connection of the anomaly to the intestine and the extent of the lesion. Thus, we decided on our surgical approach based on the CT/MRI results. Furthermore, laparoscopy could be used as a diagnostic modality rather than CT/MRI in cases with unclear ultrasound findings. In case 4 of this study, we laparoscopically explored the connection of the anomaly to the intestine.

Despite controversial operative indications, we decided on an operative indication based on the history of infection/abscess formation, or persistent symptoms. Apparently, adults are at a considerable progressive risk for cancer and might need more invasive surgery. Reportedly, UR is associated with urachal carcinoma [5, 6, 7]. Some studies have reported that pediatric patients with UR did not present with cancer in their series [5, 6]. Although urachal carcinoma is rare [5, 7], its prognosis in adults with urachal carcinoma is poor [6]. However, as the correlation between childhood UR and the development of urachal adenocarcinoma later in life is unclear [5, 7], the management of incidentally recognized asymptomatic UR is highly recommended to be resected, despite the debate.

A small UR, especially at birth, could be considered physiological. Thus, the initial management of all URs with observation is feasible in asymptomatic children [8]. Reportedly, spontaneous resolution with nonoperative management is likely with URs in patients aged <6 months [7, 9, 10]. Stopak et al. reported that the natural course of many of these URs is a spontaneous resolution, with 87% of their patients in the observation group resolving without complication within approximately one year post-diagnosis [5]. Recently, some studies reported the conservative management of UR in case reports, as well as a few small series [4, 5, 9, 11]. Nonoperative management might be extended to infected urachal cysts after the initial drainage, and adequately drained infected cysts might obliterate with time [9]. In addition, some recommend that surgical resection of patent URs be restricted to children aged >6-12 months [4, 5, 9, 11, 12]. Moreover, surgery is not without risk. Previously reported postoperative complication rates ranged from 3% to 14.7% [5, 11, 13], especially in patients aged <6 months, who accounted for 60% of wound infections [5], necessitating the consideration of routine antibiotic usage [5, 13]. In our case series, we noted a 10% complication rate (case 6) and no case recurrence. Based on our experience, the operation was performed safely even in small age groups, although the case series was small. Thus, we recommend surgical excision in patients with persistent symptoms because we experienced one case in which it was challenging to distinguish between OMDR and UR. Furthermore, we experienced four cases that required hospitalization and were administered antibiotics intravenously; of these four cases, three required drainage. Perhaps surgical excision could resolve the risk of sepsis or abscess formation.

The traditional approach to the excision of UR has been through an infraumbilical transverse or midline vertical incision. As the recently performed umbilical approach exhibited good cosmetic results, we selected the umbilical approach in our cases. Recently, although the laparoscopic approach has been demonstrated [4, 14], only a few studies have adequately defined the indication for laparoscopy [4]. Peters reported no indication for laparoscopic surgery for UR in children, except for those of uncertain origin [15]. In infants, in fact, the optimal approach would be to excise a UR from the umbilicus to the bladder dome through an umbilical incision, enabling complete excision with a smaller wound. A study reported that the achievement of a complete resection from a non-expanded umbilical incision was mostly difficult because the bladder is lower from the incision in older children [4]. In our case series, the umbilical approach exhibited good cosmetic results, and we could complete resection from the small, expanded umbilical incision even in older patients. Of note, we performed laparoscopic resection in two cases: case 4 suspected of having OMDR, and case 8 with an infected and occupied large lesion. In case 8, the omentum was tightly adhered to the abdominal wall, and the laparoscopic procedure successfully resolved the adhesion and excised the large mass lesion. In the case suspected of OMDR, the laparoscopic investigation revealed no attachment between the umbilicus and the small intestine. In cases of an umbilical cyst presenting as an OMDR, the intra-abdominal findings at laparotomy should be prioritized to eliminate any attachment to the small intestine. In addition, a complete resection of the cyst should be performed without leaving behind any residual tissue [2]. Thus, the laparoscopic approach is useful to confirm the diagnosis in cases with normal imaging findings, but recurrent umbilical symptoms should be considered, especially for older children with large lesions, previously infected cases, and suspicious OMDR cases.

The limitations of this study are that it is a small case series and a retrospective study. A larger prospective study will help establish a reasonable management plan for UR and OMDR.

## Conclusions

This study suggests that clinical examination is ideal for the initial screening of umbilical problems, such as discharge, granulation, and omphalitis. A CT or MRI could be considered when the diagnosis is unclear or complications are suspected, even though US is the first choice. Finally, surgical excision should be recommended for patients with persistent umbilical disorders, even if there is unclear evidence from diagnostic modalities.
